# Biodetection of *Mycobacterium tuberculosis*: nano-biosensors in detection; from principles to recent progresses

**DOI:** 10.1186/s13036-026-00638-9

**Published:** 2026-02-12

**Authors:** Hossein Jooya, Sama Yavari, Maryam Meskini, Seyyed Mohammad Amin Mousavi-Sagharchi, Seyed Davar Siadat, Maryamosadat Mavaei

**Affiliations:** 1 Biochemistry Group, Department of Chemistry, Faculty of Science, Ferdowsi University of Mashhad, Mashhad, Iran; 2 Department of Animal Biology, Faculty of Natural Sciences, University of Tabriz, Tabriz, Iran; 3 Microbiology Research Center (MRC), Pasteur Institute of Iran, Tehran, Iran; 4 Department of Mycobacteriology and Pulmonary Research, Pasteur Institute of Iran, Tehran, Iran; 5 Department of Microbiology, College of Basic Sciences, Shahr-e-Qods Branch, Islamic Azad University, Tehran, Iran; 6 Pharmaceutical Sciences Research Center, Health Institute, Kermanshah University of Medical Sciences, Kermanshah, Iran

**Keywords:** *Mycobacterium tuberculosis*, Tuberculosis, MTB, TB, Detection, Diagnosis, Biosensing, Biosensors, Nanobiosensors, Nanomaterials

## Abstract

Tuberculosis (TB) exerts profound detrimental impacts on global human populations. This infectious disease is primarily caused by *Mycobacterium tuberculosis* (MTB), a highly adapted pathogen that undergoes rapid genomic evolution, enabling it to acquire resistance to targeted antimicrobial agents. The emergence of chemotherapeutic resistance was first documented in the 1990s, with epidemiological data indicating that approximately 150,000 individuals succumb annually to multidrug-resistant (MDR) TB. For the detection of MTB, a variety of diagnostic approaches have been established. While culture-based methods remain the gold standard, molecular techniques offer superior reliability; however, they are costly and require specialized expertise. In the contemporary landscape of medical science, advancements in fundamental disciplines have facilitated the integration of nanotechnology into diagnostic applications, providing innovative materials for bacterial identification and infection diagnosis. Among these, nanoscale materials have been proposed as efficacious tools for MTB detection. Various nanoparticles, such as gold nanoparticles and graphene quantum dots, can be synthesized using different methodologies to enable precise identification of MTB in clinical specimens. On average, nanobiosensors achieve a limit of detection (LOD) of approximately 10³ colony-forming units per milliliter (CFU/mL), whereas magnetoelastic sensors exhibit LOD ranges from 10⁴ to 10⁹ CFU/mL. This review elucidates recent progress in nanobiosensor technologies for MTB detection, addresses associated challenges, reviews the current state of the field, and outlines prospective avenues for development.

## Introduction

Tuberculosis (TB) is a pulmonary infection that involves the lower respiratory tract. This infection is caused by a known acid-fast bacterium called *Mycobacterium tuberculosis* (MTB) [1, 2]. The affliction and mortality rate of TB are high, which has led to it being one of the most dangerous infections [3, 4]. In crowded societies (e.g., Middle Eastern countries), TB transmission is higher than in other regions. The World Health Organization (WHO) published an announcement about the end of TB in the near future, in 2030 or 2050, while drug-resistant TB (DR-TB) claims the lives of many people every day [5] WHO estimated 150 000 (95% UI: 94 000–210 000) people dead from Multi-DR (MDR) [resistance to first line of treatment]/rifampin resistant (RR)-TB in 2023 and 400 000 (95% uncertainty interval [UI]: 360 000–440 000) affliction by MDR/RR-TB. It is expected that this rate will increase in 2024 and 2025. It seems hopes for the end of TB in the near future are not possible, and DR-TB will dominate in societies as a threat [6]. Based on reports, we can conclude that the chemotherapies developed and introduced are not effective in the current situation; therefore, we need to explore further to identify novel compounds against DR-MTB.

Effective drugs have been introduced in the last ten years to fight DR-TB, and some others are in clinical trials. However, the primary therapeutic operations will launch when DR-TB is diagnosed and confirmed by laboratory methods [7]. Among all laboratory methods (e.g., culture, microscopic examination), molecular methods are the most reliable for detecting MTB with resistance properties [8]. Sequencing of MTB genomes and detection of MTB metabolites and proteins in samples are reliable methods for diagnosing TB and DR-TB. Progress in molecular sciences has led to improved diagnostic methods based on molecular details, using nucleic acid amplification (NAA) and various amplification techniques, such as polymerase chain reaction (PCR), to characterize the properties of complex pathogens, such as MTB. However, they are expensive and need adequate knowledge and expertise [9, 10]. Finding new paths to detect MTB, directly and indirectly [via antibodies], at the molecular scale is crucial for developing high-accuracy methods for TB diagnosis.

In recent decades, the emerging science of nanotechnology has been introduced and developed for diverse applications, from polymer engineering to medical sciences [11]. In medicine, nanotechnology has been used for various purposes, from drug delivery to molecular diagnosis. In diagnostics development, nanoparticles [nano-scaled materials ranging from 1 to 100 nm] [12, 13] are helpful in the detection and identification of molecules such as proteins and NAs. Also available for immunologic molecules (e.g., cytokines), NAs, and MTB surface proteins, enabling reliable detection. Optic, electrochemical, magnetic, piezoelectric, and many other types of nano-sensors have been developed to detect pathogens such as MTB in different ways [14]. MTB detection using nanobiosensors is a reliable, straightforward method for identifying molecules; it proposes the design of new, user-friendly kits for rapid TB diagnosis [15]. This review will focus on the latest achievements in nanobased detection for MTB, including mechanisms, types, and other aspects of nanobiosensing in TB. In this review, We also describe the concept of biosensing and evaluate various materials for biosensing MTB-related molecules. Furthermore, this review explores MTB molecules, their properties, and their role in nanodetection.

## Biosensing and mechanisms

Biosensors are analytical devices that generate measurable signals via an electrical or physical transducer when analytes interact with biological recognition elements, such as antibodies, nucleic acids, and aptamers [16]. These systems can detect, analyze, and transmit data from changes in biological, physiological, and chemical samples, offering a rapid alternative to conventional TB diagnosis methods such as smear microscopy and culture [17]. Unlike conventional culture methods, which can take days to yield results, nanobiosensing technologies have achieved detection times of minutes to a few hours while maintaining high sensitivity for MTB detection [18]. Since biosensors can be created in various sizes and forms, they can quantify low concentrations of biomarkers in complex compounds such as sputum or serum, thereby enabling earlier recognition. This provides the opportunity to detect infections, toxins, and early-stage pH changes [19]. Bioreceptors, transducers, and signal-processing electronics are the main components of biosensors [20]. When an MTB-specific analyte binds to the bioreceptor, a bio-recognition event occurs. The chemical changes associated with this event will be converted into an electrical, optical, or mechanical signal by a transducer, the main element in biosensors [21, 22]. The transducer translates molecular interactions into physical signals whose intensity reflects the extent of target binding [23]. After transduction, the generated signals will be amplified and then displayed or interpreted by computational modules [21]. Signal conversion in biosensors can be categorized into three main types: electrochemical, optical, and mechanical [22].

In electrochemical biosensors, quantifiable signals, such as current, voltage, or impedance, are transduced at the electrode surface via biochemical interactions [24]. The use of nanomaterials, such as gold (Au) nanoparticles and graphene (GN), enables detection at very low concentrations by increasing surface area and facilitating charge transfer, which is critical for detecting trace levels of the MTB antigen in early infection [25]. Recent advancements in graphene-based electrochemical platforms have demonstrated limits of detection as low as femtomolar and attomolar levels for antigens such as 10 kDa culture filtrate protein (CFP-10). These achievements show that electrochemical biosensors significantly surpass the sensitivity of standard enzyme-linked immunosorbent assay (ELISA)-based methods [26]. Electrochemical biosensors are widely used for their simplicity, miniaturizability, low power consumption, and high sensitivity, making them ideal candidates for developing point-of-care TB diagnostics, especially in low-resource settings [27]. This biosensor is categorized into several models depending on its transduction mechanism: amperometric, potentiometric, impedimetric, conductometric, and field-effect transistor (FET) [16]. Among these, amperometric and impedimetric biosensors are the most notable for MTB detection. Both of these systems employ various nanomaterials to enhance their sensitivity [28]. For example, an amperometric biosensor employs electrodes modified with Cadmium selenide (CdSe)/Zinc sulfide (ZnS) quantum dots (QDs) or magnetic nanoparticles [25, 29]. Likewise, an impedimetric biosensor can be integrated with nanorods, GN, or Au nanoparticles [30]. These nanocomposite-enhanced sensors demonstrate higher stability and reproducibility (RSD [relative standard deviation] < 5%), addressing the signal drift problem common in earlier-generation sensors [31, 32].

Optical biosensors capture changes that occur when an analyte interacts with an immobilized biorecognition element on the electrode surface [33]. The mechanism of detection mainly involves absorption, reflection, refraction, resonance, fluorescence, or luminescence [34]. This biosensor enables real-time, label-free detection of various molecules and targets. Additionally, high specificity, rapid analysis, cost-effectivity, and low sample requirement are other advantages of this system [35]. In optical biosensors, nanomaterials play a crucial role by enhancing the functionality through two principles. Firstly, plasmon resonance enhancement utilizes noble metal nanoparticles, such as Au, that induce a localized change in the refractive index upon target binding, dramatically shifting the surface plasmon resonance (SPR) wavelength and enabling considerable signal amplification and label-free detection [36, 37]. Secondly, quantum confinement employs QDs, such as CdSe and ZnS, as efficient fluorescent tags. QDs offer superior photostability and tunable emission spectra, making them ideal for generating a stable signal [38]. This narrow emission band enables the simultaneous detection of multiple biomarkers in a single assay [39].

Among optical biosensing techniques, SPR is valuable for its ability to monitor refractive index changes at the electrode surface during biochemical interactions [40]. This system has high initial costs; however, its reusability and compatibility with nanomaterials make it cost-effective in the long run in many aspects, such as for quantitative assessment of anti-TB medicine interactions with molecular targets and detailed evaluation of antibody-antigen binding kinetics [40]. Specifically, SPR platforms have achieved sensitivity comparable to that of molecular assays while providing critical kinetic data (kon, koff) for antibody-antigen interactions, which is unattainable with techniques such as ELISA [41]. Another optical biosensor-based system, fluorescence resonance energy transfer (FRET), can enable non-invasive detection in live cells and provide insight into intracellular MTB viability [42]. FRET is based on dipole-dipole energy transfer between donor and acceptor fluorophores, enabling real-time monitoring of conformational changes and dynamic molecular analysis [43, 44]. Electrochemiluminescence (ECL) biosensors, which combine principles of optical and electrochemical biosensors, detect target molecules by altering light emission at the electrode surface [45, 46]. This biosensor is advantageous primarily because it can identify single-base mismatches with high precision, which is essential for detecting mutations associated with drug-resistant MTB strains [47]. Compared with standard fluorescence methods, ECL can quantify MTB loads with minimal background noise due to its wide dynamic range spanning up to 6 orders of magnitude [48, 49].

Magnetic biosensors, compared to optical and fluorescent biosensors, offer more stable signals, lower background noise in biological fluids, and better-purified samples via magnetic separation [50, 51]. In this type of biosensor, detection of biomolecular interactions occurs by immobilizing recognition elements onto magnetic nanoparticles that respond to an external magnetic field, a feature particularly useful for isolating MTB from viscous sputum samples [51]. This capability allows magnetic biosensors to maintain more than 90% sensitivity even in complex, unpurified matrices where optical biosensors often fail due to light scattering or autofluorescence [52]. For MTB detection, several magnetic-based tests have been used. Magnetophoretic immunoassay (MPI), a sensitive biosensor, utilizes magnetic nanoparticles, such as magnetite (Fe_3_O_4_) or manganese ferrite (MnFe_2_O_4_) nanostructures, which can be combined with nanoparticles, such as Au nanoparticles and GN-QD, to detect antigens [30, 53]. Another magnetic-based biosensor, a magnetoresistive (MR) biosensor, can detect MTB at the single-cell level by measuring changes in electrical resistance resulting from interactions with magnetic particles [30]. Magnetic relaxation switch assays (MRSA) enable rapid, precise detection via nuclear magnetic resonance **(**NMR)-based detection of nanoparticle aggregation [54].

Piezoelectric biosensors detect analytes, such as microorganisms’ DNA and proteins, by quantifying changes in frequency due to mass [55, 56]. This label-free biosensor provides specific, real-time detection via immobilized biological elements on the crystal surface [57]. There are two main formats of Piezoelectric biosensors: quartz crystal microbalance (QCM) and series piezoelectric quartz crystal (SPQC). QCM detects changes in frequency caused by added mass on the sensor surface, enabling direct detection of whole MTB cells due to their distinct mass [55]. Although QCM is less sensitive than electrochemical methods for small molecules, this technique has achieved a LOD approximately 10^3^ CFU/mL for whole bacteria, which is comparable to smear microscopy but with higher specificity [58, 59]. This biosensor can operate in various environments, including liquids, gases, and vacuum conditions [60]. In contrast, SPQC provides more reliable, stable changes in liquid samples by detecting subtle changes in electrical conductivity rather than mass [55, 57].

## Immuno-biosensing

### Innate and adaptive immune biomarkers

**Fig. 1:**
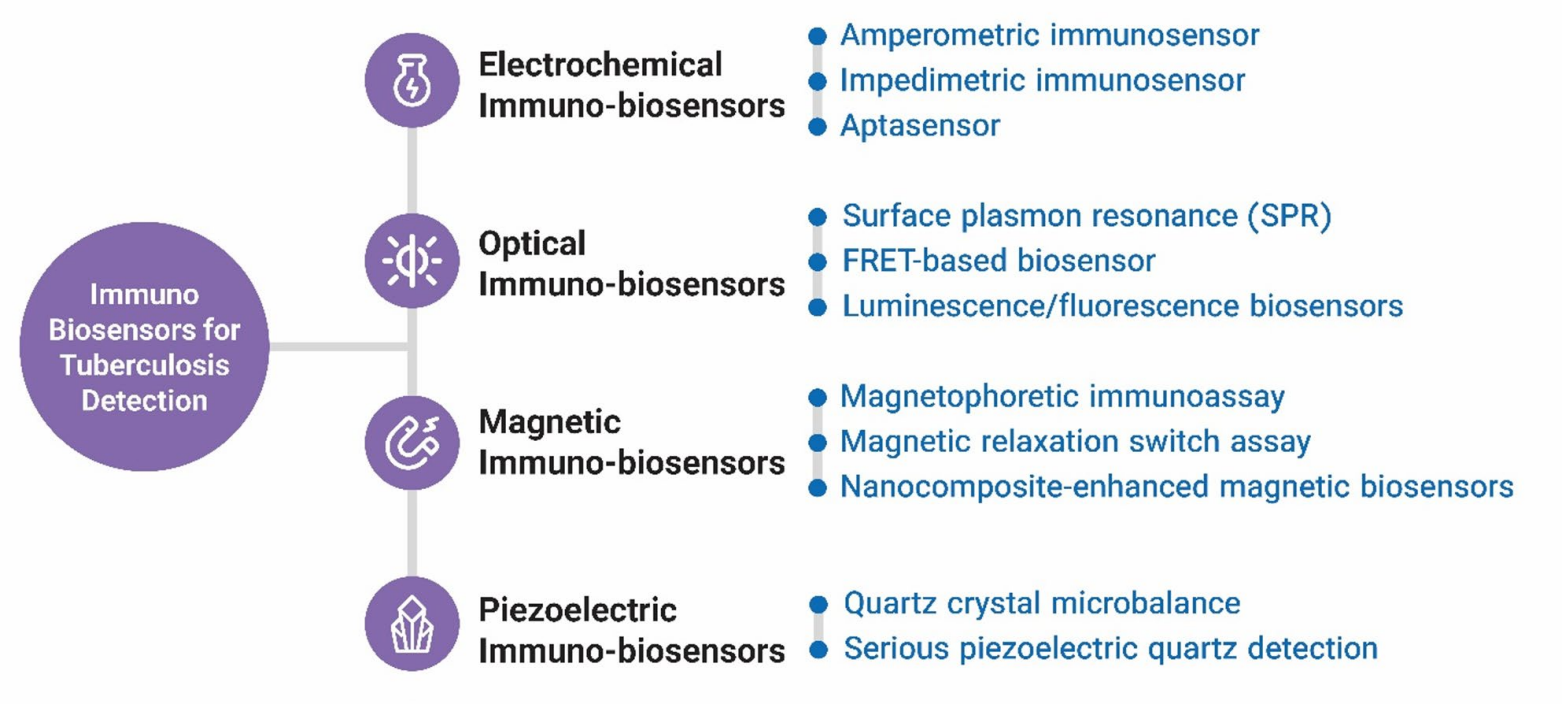
Immune-biosensing of molecules that are released in TB infection. Contains electrochemical, optical, magnetic, and piezoelectric biosensors. They were designed for the detection of immune system-released molecules in affliction to TB

Immuno-biosensing in the context of TB refers to using host immune biomarkers, including antigens, antibodies, and immune cytokines, as analytes that can be measured and transduced into signals to detect MTB [61, 62] (Fig. 1). This technique is highly valuable because MTB-caused infections directly alter the host’s immune system. The changes are tractable soon after the progression of MTB, even during latent TB infection [63, 64].

Both innate and adaptive immune responses provide valuable chemicals and molecules that biosensors can use as biomarkers. For example, macrophages are among the first cells that recognize and take up MTBs [65]. These cells release cytokines, such as interleukin (IL)-12, IL-1β, and tumor necrosis factor (TNF)-α, as well as reactive oxygen and nitrogen species, which fight MTB and serve as measurable signals [66–68]. Natural killer (NK) cells are also among the first line of defense against MTB [69]. These lymphocytes can release interferon (IFN)‐γ and IL‐22 to eliminate or suppress the invading bacteria [70]. Therefore, they serve as biomarkers for early diagnosis of TB. Additionally, IFN‐γ-induced protein (IP)-10 released by macrophages and natural killer cells is another essential biomarker that can differentiate between latent and active TB [71]. Dendritic cells that have become mature because of encountering MTBs can secrete cytokines such as TNF‐α, IFN‐α, and IL-12, which can be used as biomarkers and units that differentiate cluster of differentiation (CD)4 + T cells into T-helper (Th)-1 cells [72, 73].

In the adaptive immune response, Th-1 plays a key role in eliminating MTB, notably by triggering a cascade of immune signaling through cytokine secretion [73], including TNF-α, IFN‐γ, and IL‐2, which can serve as biomarkers for immunobiosensor detection [74, 75]. These host-derived immune molecules are the foundation of immunobiosensing, enabling TB diagnosis. Together, these host-derived immune molecules provide a valuable diagnostic target and the foundation of immunobiosensing. Moreover, early secretory target antigen six kDa (EAST-6), heat shock protein X (HspX), Hsp65, antigen 85B (Ag85B), and Ag85A are important antigens that have been extensively researched for their roles in MTB pathogenesis [76]. While EAST-6 is a crucial protein for cell biosynthesis and for activating T-cell responses, Ag85A and Ag85B also function as mycolyl transferases [76]. Overall, these antigens, along with HspX and Hsp65, can be considered critical biomarkers for diagnosing TB. Therefore, in addition to cytokines, these antigens broaden the spectrum of available biomarkers. More detailed information about the immune-derived biomarkers applied in TB biosensing is summarized in Table 1.

**Table 1:** Immunological, nucleic-acid, and protein biomarkers of MTB have been applied in biosensing technologies for TB diagnosis

Biomarker	Biomarker Type	Biosensor Strategy	Nanomaterial/Transducer	Detection Limit / Performance	Reference
IFN-γ	Immune cytokine	Electrochemical aptasensor	MOF-rGO nanocomposite	limits of detection (LOD) in pg/mL range	[77, 78]
CFP-10	MTB antigen	SPR-based immunosensor	Au thin film	Low-concentration detection, high specificity	[79, 80]
IP-10	Host cytokine	Electrochemical immunosensor	GO-modified electrode	Sub-picomolar detection reported	[81]
*IS6110*	MTB DNA	Electrochemical genosensor	rGO + AuNPs	Femtomolar detection range	[82, 83]
*mpb64*	MTB DNA	Electrochemical DNA biosensor	Carbon nanotube-modified electrode	High specificity when combined with IS6110	[84]
*rpoB* (drug resistance)	MTB DNA	RCA with magnetic nanoparticles	Magnetic nanoparticle dimerization	Dual optical & magnetic signal, mutation-specific	[85]
*katG* (drug resistance)	MTB DNA	Multiplex fluorescence quenching biosensor	QDs + nanoCoTPyP	LOD 2.0 × 10⁻⁵ µM	[86]
ESAT-6	Secreted protein antigen	Electrochemical immunosensor	Au nanoparticles, GN nanoparticles, nano-material modified electrode	Improved surface area and electron transfer rates for enhanced output	[86–88]
MPT64	Secreted protein antigen	Field-effect transistor (FET) immunosensor	GN, GN-FET	As low as 1 fg/mL real-time detection	[89]
MPT64	Secreted protein antigen	Electrochemical aptasensor	Thiolated ssDNA aptamer, interdigitated gold electrode	LOD 4.1 fM	[90]
Ag85B/MPT64	Secreted protein antigen	Optical aptasensor	GO-coated tapered optical fiber	Sub-attomolar range, detection within seconds	[91]

The table summarizes the type of biomarker, its biosensing relevance, and its application. These studies highlight how host-derived cytokines, MTB-specific antigens, and nucleic acid sequences can be exploited for sensitive and specific TB detection

In recent years, immuno-biosensing technology has evolved to detect immune biomarkers with significantly higher specificity and sensitivity. The fundamental principle behind this technology is the binding of biomarkers, such as antigens, aptamers, or cytokines, to transducers that convert these interactions into signals that can be measured and analyzed. Based on the transduction, these biosensors can be classified as electrochemical, optical, piezoelectric, and magnetic [92].

### Electrochemical immuno-biosensors

Electrochemical immunobiosensors are among the most widely studied techniques for detecting changes in electrical properties resulting from biological reactions between immune molecules and the sensor surface [93]. In addition to its advantages, such as potential for point-of-care testing (POCT), this technique’s high specificity and accuracy were demonstrated in a recent study [79]. Amperometric immunobiosensors have demonstrated high sensitivity, detecting ESAT-6, CFP10, and IFN-γ with femtomolar detection limits [94]. Aptasensors [Aptamer-based sensors] offer reliable specificity and stability, as shown in studies on detecting IFN-γ and EAST-6 using nanocomposites such as metal-organic frameworks (MOFs)/reduced graphene oxide (rGO), GN, and fullerene-polyaniline [95, 96]. These aptasensors demonstrated long-term stability, retaining more than 90% of their initial current response after storage for 4 weeks at 4 °C. This addresses a critical limitation of the antibody-based sensor [97].

Impedimetric immuno-biosensor, another electrochemical-based biosensor, has been able to detect MPT64 antigen [is encoded by the region of difference (RD)-2 region of MTB genes] in detection limits as low as 4.1 fM [1 fg/mL] and IFN-Ƴ in 0.1 picograms per milliliter (pg/mL) [98]. The use of Titanium dioxide (TiO_2_) nanorods, Au interdigitated electrodes, and 3D graphene-Au nanorods was highly effective in achieving this [98]. Although electrochemical biosensors dominate most of the research, they have some limitations that optical biosensors can address with complementary properties. This femtomolar sensitivity enables earlier diagnosis, when biomarker concentrations are below the threshold of lateral flow assays. Although electrochemical biosensors dominate most research, they have some limitations that optical biosensors can address with complementary properties.

### Optical immuno-biosensors

Among various immunobiosensors, optical biosensors offer powerful capabilities for monitoring immune biomarkers, including IFN-γ and ESAT-6. They enable rapid, non-invasive, and multiplexed detections since they resist magnetic and electrical interference [99]. Optical biosensors transduce immune-related biological interactions into measurable optical signals [100]. This happens through absorption, fluorescence, luminescence, and SPR. SPR-based immuno-biosensors were used to analyze CFP-10 and Ag85A in low concentrations with high specificity [101, 102]. This makes this group of immuno-biosensors suitable for TB diagnosis in early stages [103]. Additionally, FRET-based biosensors using QDs and Au nanoparticles can distinguish MTB from Bacillus Calmette-Guérin (BCG)-vaccinated samples with high accuracy and sensitivity by targeting ESAT-6 [104, 105]. This distinction is a significant clinical achievement, since traditional tuberculin skin tests often yield false positives in vaccinated individuals, a problem that FRET-based immune-sensors overcome.

### Magnetic immuno-biosensors

Magnetic biosensors are another efficient immuno-biosensor that can detect MTB immune biomarkers using magnetic particles in fluids such as blood and sputum. While optical biosensors are appreciated for their multiplexing and rapid results, magnetic biosensors are beneficial for their adaptability to diverse biological fluids [50]. MPI and MRSA have shown notable results in tracking biomarkers [106]. MPI detected CFP-10 at a low detection limit and high sensitivity, and MRSA detected Bacillus Calmette-Guerin (BCG) AG85 within 1 h via changes in water proton relaxation [107–109]. Incorporating these technologies with advanced-designed GN-QDs and magneto-plasmoid nanoparticles can further enhance their function and accuracy [110].

### Piezoelectric immuno-biosensors

Piezoelectric biosensors, as another promising class of immuno-biosensors, are especially valuable for their reusability. Piezoelectric immunobiosensors detect related biomarkers by measuring changes in frequency as the quartz crystal oscillates due to antigen-antibody interactions on its surface [111]. A piezoelectric technology called QCM is reusable and suitable for underdeveloped settings [58]. These technologies enable the detection of MTB using immobilized antibodies and dual detection of MTB and its antigens, such as lipoarabinomannan (LAM) [58, 112]. SPQC is a more advanced piezoelectric biosensor design. Phage-amplified is an SPQS-based biosensor that recognizes antibodies and can diagnose TB even at low concentrations [113]. Thus, by integrating various strategies, it will be possible to diagnose and control TB in low-resource settings.

## Nucleic acid-biosensing

**Fig. 2:**
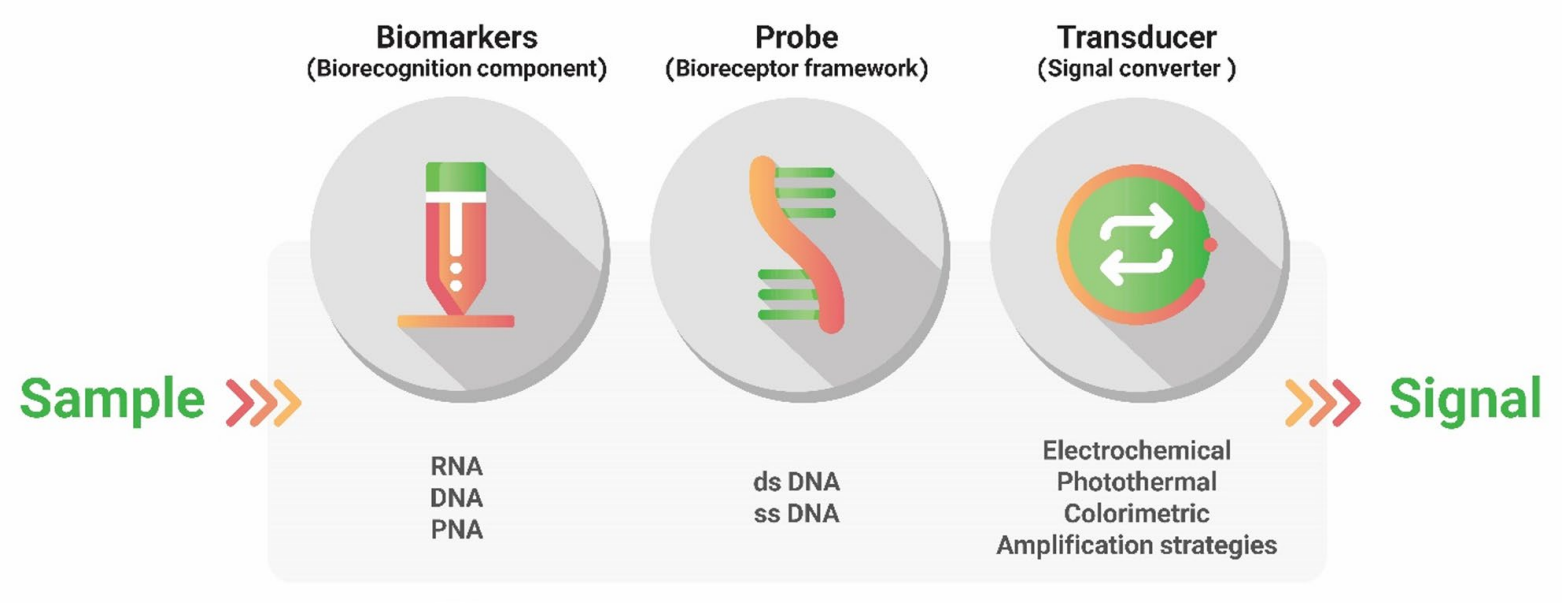
Nano-biosensor for NA detection in samples. Identification of different types of nucleic acids by probe, changing conditions, and signal production

NA biosensing refers to the use of microsystems composed of an oligonucleotide with a known base sequence or a designed NA structure incorporated or linked to a transducer [114] (Fig. 2). The majority of NA biosensors, also known as genosensors, can detect a wide range of specific DNA or RNA sequences, as well as biological or biochemical targets. This occurs through the hybridization of complementary DNA or RNA strands [115]. In more detail, the probe, designed from conserved or hypervariable regions of the genome, must be immobilized on the transducer surface. Then, if the probe detects the target NA [biomarker], the transducer [115, 116] converts its interaction into measurable signals. To obtain reliable results, it is essential to design probes to ensure they have a desirable sequence carefully. To prevent secondary structures and homology with non-target organisms in probes, using tools such as the Basic Local Alignment Search Tool (BLAST) should be considered. Unlike PCR-based methods, which require expensive thermal cycles and intensive labor, genosensors have achieved comparable sensitivity while significantly reducing the cost per test and assay time, often to less than 60 min [116, 117].

In MTB, this action ensures that biomarkers interact only with their intended targets, reducing the risk of cross-reactivity and interactions with non-target genes [118, 119]. Moreover, in NA biosensing, synthetic probes have gained attention. For example, peptide nucleic acid (PNA), due to its neutral backbone, demonstrates stronger hybridization and less non-specific binding than DNA probes, which is advantageous for working with complex samples such as blood and sputum [120, 121]. This neutral backbone eliminates electrostatic repulsion between DNA strands, allowing PNA-based sensors to maintain high sensitivity even in low-salt conditions where traditional DNA probes often fail [122]. Also, in TB, NA biomarkers play a crucial role in disease diagnosis. Insersion site *(IS)6110* is one of the most common biomarkers found in many MTB strains [123]. *Mpb64* is another targeted marker that has been used alongside *IS6110* to enhance specificity and strain coverage of biosensors [124]. Additionally, genes encoding secreted antigens such as CFP-10 and ESAT-6 are helpful for their accuracy, as they are absent from BCG vaccines [125]. Additional NA biomarkers and their applications in biosensing platforms are outlined in Table 1.

Electrochemical NA biosensors are among the most promising and widely used biosensors for detecting TB’s NAs [126]. In this technology, nanoparticles are often used to produce electrons, along with hybridization between the probe and biomarkers. Due to properties such as improved charge transfer, enhanced electrical response, and a larger surface area for the attachment of immobilized probe Au nanoparticles, conductive polymers, and GN derivatives are primarily used in these systems [24, 126]. For example, in the detection of the *IS6110* sequence, electrochemical biosensors based on nanoparticles showed significantly improved sensitivity in the femtomolar range [127]. Recent studies have shown that these nanostructured surfaces can achieve a LOD as low as 0.1 femtomolar with a broad dynamic range (1 femtomolar to 10 nanomolar) [128]. This surpasses the sensitivity of conventional gel electrophoresis [129]. To capture *IS6110*, the combination of reduced graphene oxide (GO) and Au nanoparticles added to the specificity of the electrochemical biosensor [130, 131]. Specifically, reduced GO facilitates electron transfer. It enhances the sensor’s electrochemical signal by serving as a conductive scaffold. At the same time, Au nanoparticles provide a stable platform for immobilizing a high-density probe and amplifying the signal through their catalytic activity [131]. The unique physicochemical properties of nanoparticles expanded their application beyond electrochemical biosensors. Au nanoparticles change from a dispersed state to an aggregated form due to the probe’s hybridization. This leads to changes in charge distribution and subsequent alterations in optical and thermal properties of Au nanoparticles. This is the reason why this nanoparticle is suitable for the development of colorimetric and photothermal nucleic acid biosensors [132]. For instance, in a colorimetric sensor, after binding of the *IS6110* sequence to ssDNA and the subsequent aggregation of AuNPs, the induced plasmon resonance red shift changed the suspension color from wine-red to blue. This was detectable in low concentration within one hour [133]. This visual detection enables naked-eye TB diagnosis with a LOD of approximately 10 picomolar, eliminating the need for a spectrophotometer in this setting [129]. Similarly, by using the aggregated Au nanoparticles’ photothermal effect, it was possible to detect desirable NAs via rapid temperature changes and with high sensitivity [134].

Amplification strategies are also important in NA biosensing for MTB. Since this technique replicates the NA in vitro, it can help increase the nucleic acid signal in samples with low target sequence concentration [135, 136]. PCR is a particular and sensitive amplification method, but it requires advanced thermal cycling equipment [137]. Alternatively, isothermal techniques like rolling circle amplification (RCA) and loop-mediated isothermal amplification (LAMP) offer advantages for TB POCT, as they operate at a single temperature. The amplification products are detectable in outputs such as fluorescence, electrochemical currents, or magnetic changes after integrating with nanoparticles. For example, this strategy enables rapid results without electrophoresis when PCR-amplified *IS6110* products are used as templates to generate fluorescent copper nanoparticles [138, 139].

Additionally, to identify mutations in the *rpoB* gene connected to RR, solid-phase PCR was combined with CdSe/CdS nanorods to generate detectable fluorescent signals [140]. These semiconductor nanostructures act as robust, highly efficient signal transducers and provide photostability and a narrow emission spectrum ideal for multiplexed detection in resistance gene screening [141, 142]. These nanorod-based sensors, due to their significantly reduced photobleaching, allow extended signal analysis without loss of accuracy compared to organic dyes [142]. Also, RCA with magnetic nanoparticles produces optical and magnetic signals for *rpoB* gene detection, in which the products of long, single-stranded DNA form a dimer of magnetic nanoparticles [143].

Recent developments include the use of MOF composites integrated with LAMP, which provide highly porous structures that stabilize enzymes and enhance probe localization. Therefore, this increases the detection efficiency and lowers the detection limit for MTB targets in a single-step isothermal assay [144, 145]. This MOF-LAMP integration offers ultra-sensitive detection down to single-digit copies of genomic DNA while maintaining enzymatic activity for months at room temperature, addressing related challenges in developing countries [129, 146].

## Protein targeting biosensing

MTB encodes a diverse proteome of approximately 4,000 proteins by related genes, many of which are secreted or surface-exposed and function as key virulence factors [147]. These proteins play central roles in modulating host immune responses by disrupting phagosome maturation, altering antigen presentation and cytokine expression, and influencing apoptosis and redox balance [148]. Among them, secreted antigens such as ESAT-6, CFP-10, the Ag85 complex [Ag85A/B/C], and MPT64 are particularly significant due to their diagnostic value and strong immunogenicity. These proteins play central roles in modulating host immune responses by disrupting phagosome maturation, altering antigen presentation and cytokine expression, and influencing apoptosis and redox balance [148] (Table 1).

**Fig. 3:**
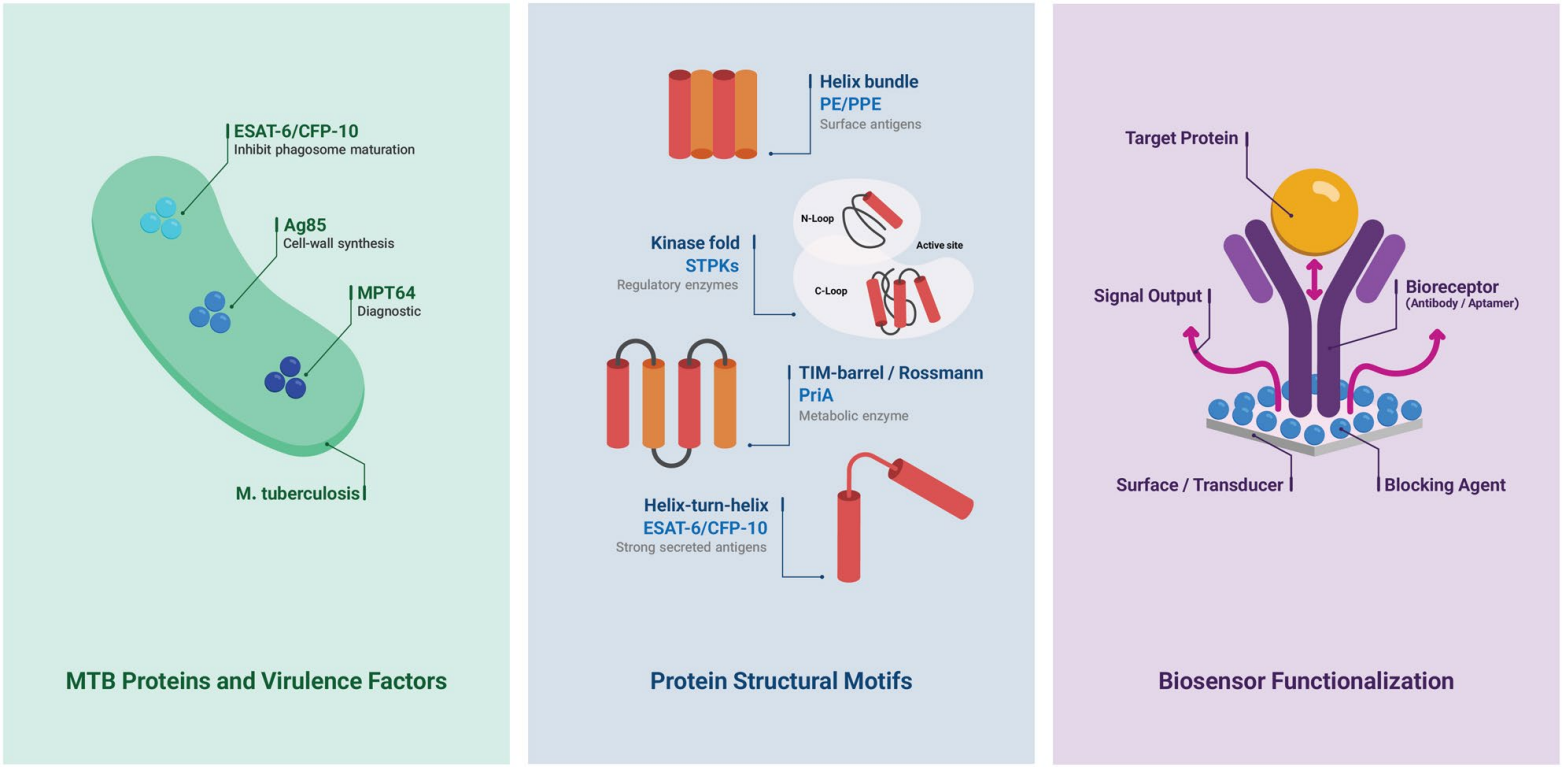
Nano-biosensing for MTB's protein detection. Various surface proteins can be used for biosensing with nanoscale sensors. The left panel illustrates key MTB proteins and virulence factors, including ESAT-6/CFP-10 (inhibiting phagosome maturation), AG85 (cell wall synthesis), and MPT64 (a diagnostic antigen). The middle panel highlights relevant protein structural motifs, including helix bundle PE/PPE (surface antigen), kinase fold STPKs (regulatory enzymes), TIM-barrrel/Rossmann PriA (metabolic enzyme), and Helix-turn-helix ESAT-6/CFP-10 (potent secreted antigens, which can serve as targets. The right panel depicts a general biosensor functionalization, in which bioreceptors (e.g., antibodies or aptamers) are immobilized on a surface/transducer. Blocking agents are then applied to cover the unoccupied surface, preventing non-specific binding from complex biological samples. This device captures the target protein (e.g., ESAT-6 or MPT64), resulting in a measurable signal.

MTB proteins exhibit considerable structural diversity, reflecting their diverse roles in pathogenicity and host interactions. These structures range from soluble helical bundles to multi-spanning membrane proteins. Most MTB proteins adopt structural motifs found in other organisms, such as the [pro-pro-glu, and pro-glu motifs] (PE/PPE) family proteins, which fold into extended α-helical bundles [149]. Similarly, serine/threonine protein kinases (STPKs) in MTB exhibit the canonical two-lobe kinase fold, with each STPK containing the complete set of 11 conserved Hanks subdomains characteristic of eukaryotic kinases [150]. Additionally, other metabolic enzymes adopt well-known structural folds, such as the TIM barrel, the Rossmann fold, and the classical kinase domain. Secreted antigen complexes, notably the ESAT-6/CFP-10 pair, form stable helix-turn-helix oligomers facilitating adequate secretion [151]. The structural integrity and specificity of these proteins are essential not only for MTB survival but also for their utility as biosensor targets, since defined structures enhance immobilization and signal reliability in biosensor platforms (Fig. 3).

In biosensor systems, the immobilization of biological recognition elements such as antibodies, enzymes, or aptamers onto the transducer surface is a critical step that directly influences sensitivity and specificity. Oriented and site-specific attachment of these bioreceptors significantly improves sensor performance. For instance, one study demonstrated that site-controlled immobilization of a lectin via bioorthogonal chemistry enhanced binding sensitivity approximately twelvefold compared to random adsorption [88]. This highlights the importance of not only the bioreceptor selection but also the method of its attachment. Upon analyte binding, the biosensor’s transducer, whether electrical, optical, or otherwise, converts the biological interaction into a measurable signal, enabling quantification of target antigen concentrations [152]. This principle underpins many current MTB diagnostic sensors, which exploit antigen–antibody or aptamer–antigen interactions to detect key MTB proteins.

To enhance biosensor performance, transducer surfaces are frequently modified using advanced nanomaterials. Noble-metal nanoparticles such as Au, silver (Ag), and platinum (Pt), as well as metal oxides such as TiO2 and zinc oxide (ZnO), are employed for their high surface-to-volume ratios, electrical conductivity, and catalytic activity, all of which contribute to increased signal output and biocompatibility. Additionally, carbon-based nanomaterials, including GNs and carbon nanotubes, and 2D semiconductors, such as transition metal dichalcogenides (TMDs) (e.g., molybdenum disulfide (MoS_2_) and tungsten disulfide (WS2)), offer excellent electrical properties and large surface areas for biomolecule immobilization. Researchers have also explored MOFs, magnetic oxide nanoparticles, and composite materials, such as metal alloys and conductive polymer–metal hybrids, further to improve electrode selectivity and stability [87]. These tailored materials enable fine-tuning of biosensor properties, including sensitivity, selectivity, and long-term operational stability, all of which are essential for clinical MTB detection. Researchers have also explored MOFs, magnetic oxide nanoparticles, and composite materials, such as metal alloys and conductive polymer–metal hybrids, further to improve electrode selectivity and stability [87].

The integration of optimized nanomaterials and specific biorecognition elements enables the construction of high-performance biosensors for MTB detection. For example, an electrochemical immunosensor targeting the ESAT-6 antigen can be developed by covalently immobilizing an anti-ESAT-6 antibody onto a nanomaterial-enhanced electrode, such as one modified with gold or graphene nanoparticles to improve surface area and electron transfer rates [87, 88]. Similarly, biosensors utilizing antibodies or aptamers against MPT64 have demonstrated impressive sensitivity. A GFET functionalized with anti-MPT64 antibodies detected antigen concentrations as low as one fg/mL in real time [89]. Another design used a thiolated single-stranded DNA aptamer co-adsorbed onto an interdigitated Au electrode, achieving a detection limit of 4.1 fM via electrochemical impedance spectroscopy [90]. Optical biosensors incorporating GN-coated tapered fibers and DNA aptamers against MPT64 and Ag85B achieved detection limits in the sub-attomolar range within seconds [91]. These examples underscore the utility of protein-targeted nano-biosensors for detecting MTB antigens with extreme sensitivity and specificity. By leveraging advances in nanotechnology and molecular recognition, such biosensors can contribute significantly to rapid, accurate, and minimally invasive TB diagnostics [30, 91].

## Whole cell detection

Whole-cell biosensors provide an alternative approach by directly capturing intact MTB bacilli rather than soluble biomarkers, preserving native antigenic structures and reducing preprocessing complexity [153]. These sensors typically use immobilized antibodies or aptamers targeting surface molecules such as LAM or ESAT-6 to bind intact bacilli [154]. The binding event is converted into quantifiable signals using platforms such as electrochemical impedance spectroscopy, FETs, or QCM, which detect physical changes, such as impedance increases or mass loading [155]. For example, QCM-based sensors detect shifts in resonant frequency due to bacterial mass, while microcantilever devices measure surface bending from cellular binding. Label-free strategies like these offer high sensitivity down to single-cell levels under optimized conditions without requiring secondary reagents. Li et al. developed a luminescent whole-cell sensor using carbon-dot-conjugated anti-LAM antibodies, reaching a detection limit of 1 × 10³ CFU/mL in under 30 min [156].

**Fig. 4:**
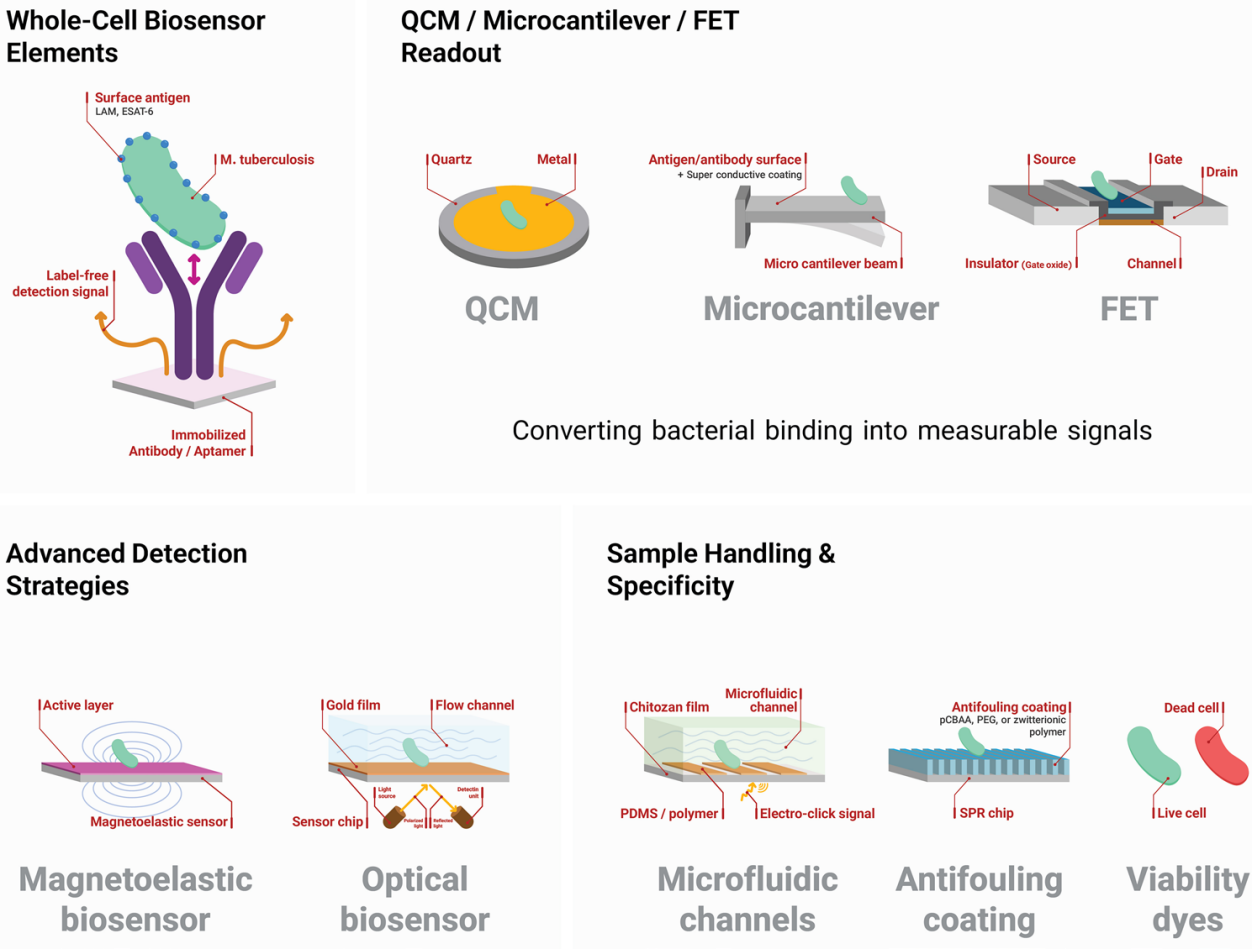
Whole-cell biosensing for MTB detection. Process of cell detection by nano-biosensors. QCM and FET, alongside advanced detection strategies such as magnetoelastic and optical biosensors, make detection easier.

Zhou et al. reported an impedimetric sensor with screen-printed electrodes and anti-LAM probes that detected 1 × 10² CFU/mL with 95% specificity in clinical sputum [154]. Complementary optical strategies, such as SPR or fluorescence-based systems, have also enabled real-time visualization of bacterial capture. Magnetoelastic biosensors with wireless readouts have been used to monitor bacterial metabolism by detecting changes in media conductivity or viscosity, achieving detection thresholds of 10⁴ CFU/mL [157]. Significantly, the clinical significance of these values ​​varies. At the same time, sensors with thresholds near 10⁴ CFU/mL offer sensitivity comparable to that of conventional smear microscopy. Those that achieve a range of 50–100 CFU/mL meet the WHO target product profiles for high-sensitivity triage tests and potentially detect pseudobacillary infections missed by traditional microscopy [158]. To address matrix complexity, microfluidic handling improves wash and concentration efficiency, while antifouling coatings, such as polyethylene glycol, zwitterionic polymers, or hydrogel films, reduce nonspecific binding and preserve capture sensitivity in viscous samples, such as sputum [159, 160]. Viability dyes further differentiate live versus dead cells by modulating signal fluorescence, improving clinical interpretability [161]. Collectively, whole-cell biosensors bypass nucleic acid amplification, enabling direct, rapid, and point-of-care MTB detection with clinically meaningful performance (Fig. 4).

## Comparison of biosensors and their performance

Comparative evaluations of biosensor platforms for MTB show that transduction principles fundamentally determine the trade-offs between sensitivity, assay duration, and operational complexity [162]. Electrochemical biosensors, encompassing amperometric, potentiometric, and impedimetric formats, remain the most widely developed due to their low fabrication costs, potential for miniaturization, and portability. Using conductive electrodes to convert target binding into electrical signals, they achieve LODs between 10¹–10³ CFU/mL within 30–60 min [154, 163]. Advanced nanostructured electrode designs have extended performance to femtomolar protein levels, though they require rigorous calibration and noise filtering for reproducible results [89, 164]. FETs functionalized with nanomaterials such as graphene deliver sub-femtogram-per-milliliter sensitivity for antigens like MPT64 in under 15 min, offering unmatched speed for point-of-care applications [89, 165]. However, both electrochemical and FET platforms face challenges in scaling production and maintaining performance in complex biological matrices [163, 166].

Optical biosensors, including fluorescence, chemiluminescence, SPR, and optical fiber devices, enable label-free and real-time detection with high specificity [91]. They typically achieve LODs of around 10³ CFU/mL for whole cells or proteins, with specific advanced configurations, such as GO-enhanced tapered optical fibers functionalized with DNA aptamers, reaching attomolar sensitivity for targets such as MPT64 and Ag85B [91]. Multiplexing capability makes them appealing for simultaneous biomarker detection, yet the complexity, cost, and instrument size limit their deployment in resource-limited settings [164]. Overcoming these barriers will require miniaturization and integration with low-power optical components without compromising analytical performance.

Piezoelectric platforms, such as QCM and cantilever systems, detect direct mass changes upon bacterial adhesion or biomolecular interaction. In optimized laboratory settings, they have achieved LODs as low as 10² CFU/mL and even single-cell detection. However, their performance is strongly influenced by temperature, sample viscosity, and microbubble formation, necessitating strict environmental control [153, 165]. Magnetoelastic sensors, while mechanically related, monitor shifts in resonant frequency of magnetically active films and offer wireless, low-power operation. These devices have detected MTB cultures in the 10⁴–10⁹ CFU/mL range, making them robust and field-deployable but less sensitive than molecular or advanced immunosensing methods [157].

**Fig. 5:**
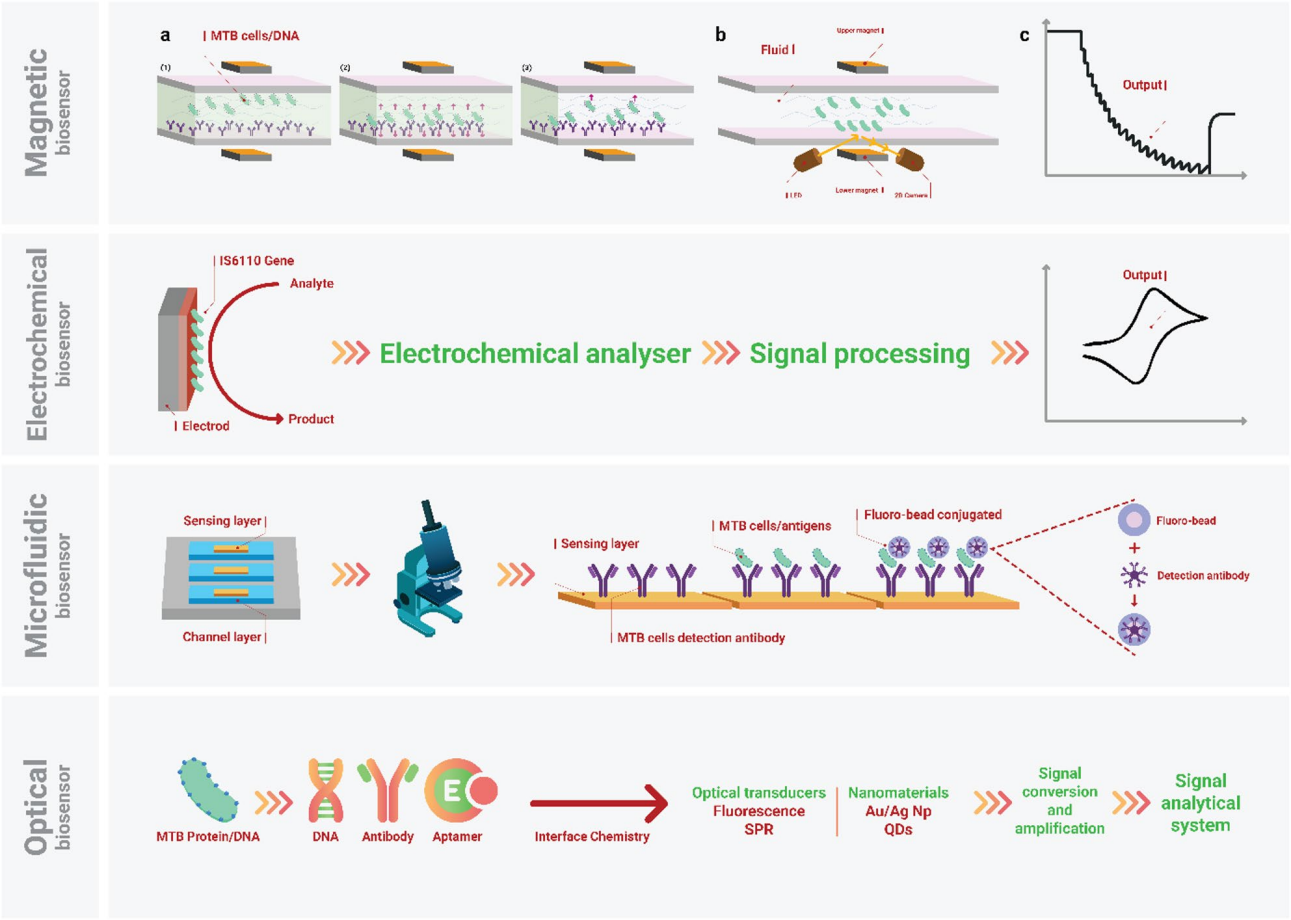
Comparison of four distinct nano-biosensors for MTB detection: Magnetic biosensor: Depicts the detection of MTB cells or DNA in complex fluid. External magnetic fields isolate the target by manipulating magnetic particles, resulting in a measurable change in optical signal or magnetic relaxation due to particle aggregation. **Electrochemical biosensor**: Illustrates the diagnosis of TB by detecting the NA marker (e.g., the *IS6110* gene). The binding of the analyte to the electrode surface initiates a redox reaction that produces a quantifiable electrical signal. **Microfluidic biosensor**: demonstrates a chip-based platform where MTB cells/antigens are captured on the surface by MTB cell detection antibody. Signals are generated through a sandwich format using a detection antibody conjugated to a fluorescent microbead. This enables optical results. **Optical biosensor**: MTB protein/DNA binds to immobilized biorecognition elements. This binding then converts to a measurable signal via transduction methods such as fluorescence or SPR, enabling optical detection

Magnetic-particle-based systems integrate rapid target enrichment with microfluidic detection, achieving LODs as low as 10¹ CFU/mL and enabling multiplex assays. High-gradient magnetic fields concentrate pathogens quickly, reducing assay times and improving specificity in complex samples [163]. Despite these advantages, reliance on power-intensive magnets and precise fluidic control complicates portable operation. Aptamer-based sensors represent another emerging strategy, with synthetic nucleic acid ligands offering superior thermal stability compared to antibodies and reaching LODs near 10² CFU/mL in electrochemical formats. Nevertheless, their slower binding kinetics and need for careful sequence optimization can limit clinical throughput [167]. Strategic integration of complementary modalities, such as combining magnetic enrichment with electrochemical or optical transduction, may help overcome existing trade-offs in sensitivity, speed, and operational simplicity [167]. Table 2; Fig. 5 describe differences among nano-biosensors, their mechanisms, advantages, and limitations, which are applicable for MTB detection.

**Table 2:** Evaluation and comparison of different materials in nano-biosensing of MTB

Biosensor Platform	Transduction Principle	Typical LOD	Assay Time	Key Advantages	Key Limitations	Ref
Electrochemical Immunosensors	An electrical signal from the target binding to the electrodes	10¹–10³ CFU/mL; femtomolar proteins	30–60 min	Low cost; miniaturization; portability; high sensitivity; Ultra-high sensitivity.	Requires rigorous calibration, noise filtering, and complex matrices.	[89, 154, 163]
FETs	Conductance modulation by biomolecular interaction	Sub-fg/mL for proteins (e.g., MPT64)	< 15 min	Ultra-high sensitivity; rapid response; point-of-care use.	High fabrication cost; specialized microfabrication.	[89, 166]
Optical (fluorescence, SPR, fiber)	Optical property changes upon binding	~ 10³ CFU/mL; attomolar for specific antigens	Real-time	Label-free detection, high specificity, multiplexing.	High cost; bulky instrumentation; limited field use.	[157, 164]
Piezoelectric (quartz crystal, cantilever)	Frequency/mass change upon target binding	10² CFU/mL; single-cell sensitivity	Rapid	Direct label-free mass sensing; simple interpretation.	Sensitive to temperature/viscosity; requires control.	[153, 165]
Magnetoelastic Sensors	Resonant frequency shift in magnetically active film	10⁴–10⁹ CFU/mL	Rapid	Wireless, low-power, field robust.	Low sensitivity compared to molecular methods.	[167]
Magnetic-Particle-Based Microfluidics	Magnetic enrichment combined with on-chip detection	10¹ CFU/mL	Rapid	High specificity; multiplexing.	Power-intensive magnets; precise fluidic control needed.	[163, 166]
Aptamer-Based Electrochemical Sensors	Target binding to nucleic acid ligands	10² CFU/mL	Moderate	High thermal stability; antibody-free.	Slower kinetics; sequence optimization required.	[153]

Key advantages and limitations are described alongside the principal concepts

## Digital microfluidics and AI in MTB detection

To overcome the sensitivity limitations of volumetric measurements in paucibacillary TB, recent research has increasingly focused on microfluidic digital biosensing. This approach involves dividing sputum or plasma samples into thousands of discrete microreactions, enabling the absolute quantification of DNA or MTB antigens (e.g., ESAT-6) at the single-molecule level [168, 169]. By isolating target biomolecules in femtoliter-sized compartments, these platforms minimize background interference from complex sputum matrices, thereby significantly increasing the signal-to-noise ratio for MTB detection [170]. PCR-related approaches assist us in design useful digital microfluidics. Microfluidics-based digital PCR consists of two main groups: (1) droplet-based digital PCR (ddPCR), and (2) chamber-based digital PCR (cdPCR). In comparison between them, cdPCR microfluidics has demonstrated more accuracy than ddPCR in application. Steps in cdPCR are as follows: (1) PCR reaction mixture is diffused in a very small scale (1/100 of reaction units [reaction chambers]); (2) Molecules are amplified, and huge amounts of copies are prepared; (3) Accurate quantifications of copies of NAs are achieved by detecting emitted fluorescence signals. (4) Poisson distribution statistics is used for calculation of sample’s copy number [171, 172]. In this regard, a recent pivotal review by Tong et al. emphasized the development of cdPCR devices. It demonstrated that precise microfluidic design is essential for high-throughput (HT) fractionation to detect rare MTB targets with accuracy comparable to culture methods, but in a fraction of the time [173]. Such digital strategies effectively bridge the gap between rapid screening and the precise quantification required to monitor treatment response in TB patients [174].

Complementing these hardware innovations, the integration of artificial intelligence (AI) into nanobiosensor platforms is revolutionizing the interpretation of MTB diagnostic data [175]. Machine learning (ML) algorithms and deep learning (DL) models have been employed to analyze complex signals from colorimetric, plasmonic, or electrochemical MTB sensors, distinguishing true-positive signals from non-specific binding noise with high accuracy [176, 177]. For example, AI-assisted image processing can automate the reading of smartphone-based fluorescence microscopes or lateral flow assays, thereby reducing human error in resource-limited settings [178]. This synergy between microfluidic digitization and AI-based analysis will not only improve MTB detection limits but also facilitate the development of portable sample-to-response devices that meet the WHO requirements for next-generation TB POCT [179].

## Current challenges and future perspectives

Despite notable laboratory advances, MTB biosensors still face critical barriers to clinical translation. Sample preparation remains a primary bottleneck, as complex clinical matrices such as viscous sputum and blood require pretreatment steps, including liquefaction, dilution, antifouling, or filtration to minimize matrix effects and interfering substances, thereby improving reproducibility [160, 180]. The current lack of standardized protocols for diluents and antifouling agents contributes to variability in diagnostic performance across platforms [162]. Stability and shelf-life are further constraints; degradation of biological recognition elements, such as antibodies or aptamers, over time, and batch-to-batch variability of nanomaterial-based electrodes can reduce accuracy and limit deployment in resource-limited settings [181, 182]. Another significant challenge is the limited multiplexing capacity of current devices, which typically target only a single biomarker. Simultaneous detection of multiple antigens or cytokines could substantially improve diagnostic accuracy, enable disease staging, and guide treatment monitoring [183]. Integration of microfluidics for on-chip sample processing, fluid control, and automated readout offers a potential solution for assay complexity, yet increases fabrication costs and demands interdisciplinary engineering [159]. In this context, ensuring reproducible manufacturing and maintaining performance under variable field conditions are crucial for point-of-care applicability.

Emerging innovations point toward highly integrated, low-cost, and portable formats. Wearable or paper-based biosensors promise ultra-low-cost decentralized TB screening, although achieving reproducible quantitative readouts remains a challenge [184]. Coupling biosensors with smartphone-based optical or electrochemical readouts could facilitate field deployment, while integrating with AI-driven signal processing could enhance the interpretation of complex outputs. Such AI algorithms require large, well-annotated datasets for robust model training, which are currently scarce in TB diagnostics [185]. Novel transduction schemes also expand possibilities; for example, an “electronic nose” combining piezoelectric sensors with volatile metabolite analysis has demonstrated rapid MTB culture detection with detection limits approaching 10 cells/mL in multichannel configurations [186].

While several comprehensive reviews, such as those published by Pourakbari et al. (2019) [187] and Golichenari et al. (2019) [188], have provided excellent summaries of MTB biosensors based primarily on individual transduction mechanisms (e.g., electrochemical versus optical detection), a significant gap remains in assessing their system-level portability and integration readiness [189]. This review distinguishes itself by providing a structured analysis that links fundamental principles of nanomaterial design with critical requirements for POC detection [190]. We uniquely focus on the convergence of nanobiosensing with digital microfluidic platforms and AI-assisted signal processing, and evaluate these integrated systems against WHO Targeted Product Profiles (TPPs) [191]. This approach provides a more precise roadmap for moving research prototypes into reliable clinical tools, which is the ultimate differentiator of this work [192].

Unlike previous reviews that categorized biosensors solely by transduction mechanism (e.g., optical versus electrochemical), this review offers a new perspective by assessing the “system-level readiness” of these platforms. We explicitly categorize recent advances based on their integration with digital microfluidics and AI-based analytics, highlighting this convergence as a critical path to bridging the gap between benchtop sensitivity and clinical application [190]. Ultimately, addressing these technical and practical challenges through robust surface chemistries, hardware integration, and rigorous clinical validation will be essential to transition nano-biosensor platforms from research prototypes to reliable diagnostic tools for global TB control [193, 194].

## Conclusion

MTB, as one of the cryptic bacteria, can infect human lungs and cause one of the primary fatal human diseases. TB is diagnosed when an infected human shows signs and symptoms. Prognosis systems for detecting bacterial cells are weak and need improvement. Nanotechnology, as a pioneering science in different fields, can help us in medicine. Nano-scale materials can be used as detectors of various molecules. Different materials, from metals to organic materials, can be used as nano-biosensors. Also, various methods, such as detection of cellular and molecular elements [e.g., genes, proteins [superficial and secretory], produced immunoglobulins and cytokines, etc.], are available for direct and indirect detection of MTB. Different platforms have been designed to realize this purpose. Optical, electrochemical, piezoelectrical, and magnetic platforms are the leading platforms for detecting different elements of MTB, each with its own strengths and weaknesses. Magnetic-particle-based microfluidics is a high-specificity platform, and FET is an ultra-high-sensitivity platform. High accuracy and rapid presentation of results are the main advantages of nanobiosensing platforms.

Diagnosis and prognosis of TB are the main factors in disease control and eradication. Nanobiosensors can launch these functions, and their handling could pave the way for a bright future in TB control. TB control programs for the central afflicted countries that are situated in high-burden line of TB prevalence have been proposed by WHO. Programs have been planned to end TB in 2030 and 2050; however, DR-TB is the main disaster to reach this dream [world without TB]. Nanotechnology emerges with the ability to detect MTB at the molecular level. The development of crafted tools made from nanomaterials can better arm humans than in the past to fight TB.

## Data Availability

All data are available within the manuscript.

## Abbreviations

MTB: Mycobacterium tuberculosis
TB: Tuberculosis
WHO: World Health Organization
DR: Drug resistance
MDR: Multi-DR
XDR: Extensively DR
NAA: Nucleic acid amplification
PCR: Polymerase chain reaction
FET: Field-effect transistor
CdSe: Cadmium selenide
ZnS: Zinc sulfide
QDs: Quantum dots
SPR: Surface plasmon resonance
FRET: Fluorescence resonance energy transfer
ECL: Electrochemiluminescence
MR: Magnetoresistive
MRSA: Magnetic relaxation switch assays
QCM: Quartz crystal Microbalance
SPQC: Series Piezoelectric quartz crystal
IFN-γ: Interferon gamma
IL: Interleukin
TNF: Tumor necrosis factor
EAST-6: Early secretory target antigen
Hsp: Heat shock protein X
CFP10: 10-kDa culture filtrate protein
MOF: Metal-organic framework
GN: Graphene
rGO: Reduced graphene oxide
TiO_2_: Titanium dioxide
TMDs: Transition metal dichalcogenides
MoS_2_: Molybdenum disulfide
WS2: Tungsten disulfide
BLAST: Basic Local Alignment Search Tool
RCA: Rolling circle amplification
STPKs: Serine/threonine protein kinases
LAM: Lipoarabinomannan
Au: Gold
Ag: Silver
Pt: Platinum
ZnO: Zinc oxide
LODs: Limits of detection
AI: Artificial intelligence
ML: Machine learning
DL: Deep learning
